# Time trends of cancer mortality among elderly in Italy, 1970–2008: an observational study

**DOI:** 10.1186/1471-2407-12-443

**Published:** 2012-10-02

**Authors:** Ettore Bidoli, Lucia Fratino, Silvia Bruzzone, Marilena Pappagallo, Paolo De Paoli, Umberto Tirelli, Diego Serraino

**Affiliations:** 1 Unit of Epidemiology and Biostatistics, Centro di Riferimento Oncologico, IRCCS, via Franco Gallini 2, 33081 Aviano, PN, Italy; 2 Division of Medical Oncology A, Centro di Riferimento Oncologico, IRCCS, Aviano, Italy; 3 Direzione Centrale per le Statistiche e le Indagini Sulle Istituzioni Sociali, Servizio Sanità e Assistenza, National Institute of Statistics, Rome, Italy; 4 Direzione Scientifica, Centro di Riferimento Oncologico, IRCCS, Aviano, Italy; 5 Department of Medical Oncology, Centro di Riferimento Oncologico, IRCCS, Aviano, Italy

**Keywords:** Cancer mortality, Time trends, Elderly, Italy

## Abstract

**Background:**

The aging of the Italian population will unavoidably lead to a growing number of persons diagnosed and living with cancer. A comprehensive description of the burden of cancer mortality among Italian elderly (65-84 years of age) in the last four decades has not been carried out yet. Cancer mortality rates were used to describe time trends between 1970-2008.

**Methods:**

Mortality counts, provided by the Italian National Institute of Statistics, were grouped according to data availability: in quinquennia from 1970-74 through 1995-99, and in 2000-03 and 2006-08 groups. Age-standardized rates (world population) were computed by calendar periods while annual percent changes (APCs) were computed for elderly and middle aged (35-64 years) people for the period 1995-2008.

**Results:**

The number of cancer deaths in elderly nearly doubled between 1970-74 (31,400 deaths/year in men, and 24,000 in women) and 2006-08 (63,000 deaths/year in men, and 42,000 in women). Overall cancer mortality rates peaked during the quinquennia 1985-89 and 1990-94 (about 1,500/100,000 in men and 680 in women) and declined thereafter. Throughout 1995-2008 cancer mortality rates decreased by -1.6%/year in men and -0.9%/year in women. These decreases were mainly driven by cancers of the stomach, bladder, prostate, and lung (APC = -3.3%, -2.7%, -2.5%, -2.2%, respectively) in men, and by cancers of the stomach, bladder, and breast (APC = -3.5%, -1.9%, -1.1%, respectively) in women. Conversely, increases in mortality rates between 1995 and 2008 were recorded for lung cancer (APC = +0.6%) in women, cutaneous melanoma (APC = +1.7%) in men, and pancreatic cancer (APC = +0.6% in men and +0.9% in women).

**Conclusions:**

Overall favorable trends in cancer mortality were observed among Italian elderly between 1995 and 2008. Early diagnosis, improved efficacy of anti-cancer treatments and management of comorbidities are the most likely explanations of these positive observations. However, enduring preventive interventions against the most common risk factor (e.g. cigarette smoking), early diagnosis, and access to care should be reconsidered and extended to match the reductions of cancer mortality recorded in the elderly with those in the middle aged.

## Background

It is well known that the incidence of most cancers increases approximately as the 4^th^-5^th^ power of age. However, aging should not be considered an independent risk factor but a surrogate of prolonged carcinogen exposure [1-3]. In 2008, in Italy, nearly 60% of about 150,000 cancer deaths occurred in the 10.5 million elderly aged between 65 and 84 years. Elderly population is expected to increase by 11% in 2020, and by 29% in 2030 [4], and this increase will unavoidably lead to a growing number of elderly people diagnosed and living with a neoplastic disease.

Mortality rates from some neoplasms steadily declined among the whole Italian population up to the last published update in 2007 [5,6]. Downward trends were mainly recorded for cancers of the stomach and intestines in both sexes, and in men only, for lung and other tobacco-related neoplasms. For some cancer types it is likely that the widespread implementation of early diagnosis programs (e.g. breast and prostate) and/or the introduction in the clinical practice of new treatments (e.g. testis, Hodgkin’s lymphoma, or lymphocytic leukemias) contributed to the reduction of cancer mortality.

In the elderly the behavior of some cancers may become more aggressive (e.g., acute myeloid leukemia, lymphoma and ovarian cancer) or more indolent (e.g., breast and lung cancer) than in the adult age groups [7,8]. From this view point, the assessment of long term trends of cancer mortality offers specific clues about the introduction of early diagnosis modalities, the development of new treatment protocols, and competing causes of mortality (e.g., cardiovascular and neurologic disorders). A topic that has been poorly investigated in Italy [7,8].

In the very elderly (≥85 years), the identification of the precise cause of death is potentially complicated by a number of common life-threatening comorbidities (i.e. hypertension, chronic obstructive pulmonary diseases, or diabetes). Thus, in order to reach meaningful conclusions, analysis was restricted to the elderly.

We took advantage of the availability of computerized death certificates recorded in Italy over the 1970-2008 period to describe trends in cancer mortality among the elderly aged 65-84 years.

## Methods

Information regarding the underlying cause of death for the period 1970–2008 was extracted from the database available on electronic support from the Italian National Institute of Statistics (ISTAT) [4]. Between 1970 and 2008, three different revisions of the International Classification of Disease (ICD) were used to code the underlying cause of death: the 8th revision from 1970 to 1979 [9]; the 9th revision from 1980 to 2002 [10]; and the 10th revision since 2003 [11]. To improve validity and comparability of some cancer sites/types throughout subsequent classifications, we pooled together all oral cavity and pharynx neoplasms, all intestinal sites, including rectum, all non-Hodgkin’s lymphomas, and all leukemias. Deaths were recorded according to the ICD-10 as: all causes of death (ICD10: A00-Y99), all malignant and benign cancers (ICD-10: C00-D48), lip, oral cavity and pharynx (ICD10: C00–C14), esophagus (ICD-10: C15), stomach (ICD-10: C16), intestines, mainly colon and rectum (ICD-10:C18–C21), liver (ICD-10:C22.0, C22.2, C22.3, C22.4, and C22.7), pancreas (ICD-10: C25), larynx (ICD-10: C32), lung (ICD-10:C33–C34), cutaneous melanoma (ICD-10:C43), prostate (ICD-10: C61), breast (ICD10: C50), ovary (ICD-10:C56), bladder (ICD-10: C67), kidney and other urinary sites (ICD-10: C64–C66, C68), all leukemia (ICD10: C91–C95), Hodgkin’s lymphoma (ICD10:C81) and non-Hodgkin’s lymphomas (ICD-10: C82–C85, C96). Resident population by sex, age, and calendar year was abstracted from the ISTAT database [12].

Data were grouped into quinquennia from 1970-74 to 1995-99, and into two shorter periods (2000-2003 and 2006-2008), because the computerization of death certificates for the years 2004 and 2005 had not been completed at the time of data analysis. Age-standardized mortality rates per 100,000 (to the world standard population [13]) were calculated in both sexes by grouped periods and annually between 1995 and 2008 according to the underlying cause of death. Rates and their corresponding standard errors were calculated using SAS 9.20 (SAS Institute Inc., Cary, NC).

The computation of annual percent change (APC) [14,15] of mortality rates was restricted to the 1995-2008 period to specifically quantify the recent impact of cancer mortality on the elderly and on the middle aged (35-64 years) populations. APCs were estimated by fitting a linear regression line to the natural logarithm of annual mortality rates using calendar year as a regressor variable. This calculation assumes that the mortality rates changed at a constant rate over the entire calendar-year interval examined, and the validity of this assumption was checked by merely examining plotted curves. Statistical significance was assessed at p < 0.05.

## Results

The number of cancer deaths and the proportion of deaths caused by cancer nearly doubled during the study period among the Italian elderly population (Table 1). In particular, the proportion increased, in men, from 21.5% in 1970-74 (31,400 deaths per year from any cancer) to 39.8% in 2006-08 (63,000 deaths/year), and from 16.8% (24,000 deaths/year) to 32.7% (42,000 deaths/year) in women. In 2006-2008, half of overall cancer mortality in the elderly was explained, in both sexes, by few leading cancers, namely: lung (27.9%); intestines (10.3%); prostate (7.6%) and stomach (6.3%) in men, and breast (13.8%); intestines (11.8%); lung (10.7%); pancreas (7.5%) and stomach (5.8%) in elderly women (Table 1).

**Table 1:** Number of deaths of selected cancers by sex according to 1970-74 and 2006-08 periods among elderly (65-84 years) population; Italy 1970-2008

**Cause of death or groups of diseases**	**Period**			
	**1970-74**		**2006-08**	
	**Mean number of deaths per year in 65-84 age group**	**% on all: deaths**^**+**^**, cancers°**	**Mean number of deaths per year in 65-84 age group**	**% on all: deaths**^**+**^**, cancers°**
Men				
All causes of death	146502	-	158647	-
All cancers, benign and malignant	31435	21.5+	63160	39.8+
Lip, oral cavity and pharynx	1010	3.2°	1001	1.6°
Esophagus	887	2.8°	783	1.2°
Stomach	6052	19.3°	3981	6.3°
Intestines, mainly colon & rectum	3400	10.8°	6498	10.3°
Liver	541	1.7°	1757	2.8°
Pancreas	934	3.0°	3030	4.8°
Larynx	1045	3.3°	944	1.5°
Lung	6551	20.8°	17639	27.9°
Cutaneous melanoma	54	0.2°	477	0.8°
Prostate	2898	9.2°	4824	7.6°
Bladder	1499	4.8°	2924	4.6°
Kidney	366	1.2°	1539	2.4°
Leukemia	787	2.5°	2015	3.2°
Hodgkin lymphoma	159	0.5°	113	0.2°
Non-Hodgkin lymphoma	293	0.9°	1497	2.4°
Women				
All causes of death	142098	-	127897	-
All cancers, benign and malignant	23911	16.8+	41839	32.7+
Lip, oral cavity and pharynx	214	0.9°	384	0.9°
Esophagus	253	1.1°	245	0.6°
Stomach	4694	19.6°	2434	5.8°
Intestines, mainly colon and rectum	3478	14.5°	4935	11.8°
Liver	495	2.1°	448	1.9°
Pancreas	856	3.6°	3138	7.5°
Larynx	65	0.3°	79	0.2°
Lung	1144	4.8°	4465	10.7°
Cutaneous melanoma	54	0.2°	321	0.8°
Breast	2842	11.9°	5781	13.8°
Ovary	558	2.3°	1764	4.2°
Bladder	415	1.7°	638	1.5°
Kidney	255	1.1°	762	1.8°
Leukemia	634	2.7°	1487	3.6°
Hodgkin lymphoma	125	0.5°	97	0.2°
Non-Hodgkin lymphoma	237	1.0°	1384	3.3°

**Figure 1:**
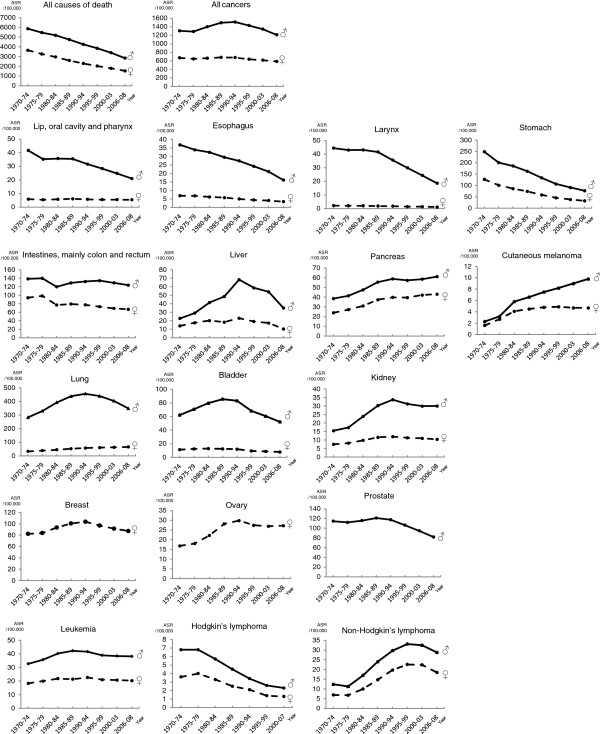
**Trends of age-adjusted mortality rates (ASR) (world population) of cancers and non-cancers among elderly (65-84 years) population. **Italy, 1970-2008.

During the study period, overall cancer mortality rates peaked during the quinquennia 1985-89 and 1990-94 in both sexes and declined thereafter (Table 2 and Figure 1). In elderly men, rates increased from 1,306/100,000 in 1970-74 to nearly 1,500 during the quinquennia 1985-89 and 1990-94, and then dropped to 1,212 in 2006-08. In elderly women, the rates increased from 671/100,000 in 1970-74 to nearly 680 during the quinquennia 1985-89 and 1990-94, and then dropped to 586 in 2006-08. Between 1995-2008, APC decreased by -1.6%/year (95%CI: -1.8;-1.4) in men, and by -0.9%/year (95%CI: -1.0;-0.7) in women. A large variability in cancer trends was registered according to sex, period and site/type.

**Table 2:** Time trends of selected cancer death rates among elderly and middle aged populations; Italy, 1970-2008

**Cause of death or groups of diseases**	**Age Standardized Rates (World population) in elderly (65-84 years) x 100,000**								**Annual Percent Change from 1995 to 2008**			
	**Period**								**Elderly (65-84 yrs)**		**Middle aged (35-64 yrs)**	
	**1970-74**	**1975-79**	**1980-84**	**1985-89**	**1990-94**	**1995-99**	**2000-03**	**2006-08**	**APC**	**(95% CI)**	**APC**	**(95% CI)**
Men												
All causes of death	5876,5	5478,3	5193,2	4750,8	4262,2	3857,5	3397,7	2847,8	-2,9*	(-3,1;-2,7)	-3,1*	(-3,2;-2,9)
All cancers, benign and malignant	1305,8	1288,1	1400,6	1495,6	1511,6	1431,1	1343,9	1211,5	-1,6*	(-1,8;-1,4)	-2,6*	(-2,6;-2,5)
Lip, oral cavity and pharynx	41,6	35,2	35,7	35,5	31,6	28,3	24,8	21,0	-2,8*	(-3,3;-2,3)	-3,2*	(-3,8;-2,7)
Esophagus	36,8	33,9	32,4	29,5	27,4	24,2	21,1	16,1	-3,7*	(-4,6;-2,9)	-4,2*	(-4,8;-3,6)
Stomach	248,8	201,0	186,2	162,7	134,0	106,7	91,3	76,8	-3,3*	(-3,6;-3,0)	-4,0*	(-4,4;-3,7)
Intestines, mainly colon and rectum	138,5	139,8	119,9	128,9	132,3	134,2	129,1	123,7	-0,8*	(-1,0;-0,6)	-1,2*	(-1,5;-0,8)
Liver	22,8	29,1	41,4	48,5	68,3	58,7	54,0	35,0	-4,6*	(-6,8;-2,4)	-6,1*	(-8,1;-4,2)
Pancreas	38,6	41,4	47,6	55,5	58,8	57,3	58,5	61,1	+0,6*	(+0,4;+0,9)	+0,1	(-0,3;+0,4)
Larynx	44,4	43,0	43,1	41,6	35,6	29,9	24,4	18,6	-4,6*	(-5,1;-4,0)	-4,5*	(-5,5;-3,5)
Lung	282,7	329,7	392,0	438,4	456,6	439,4	403,7	347,8	-2,2*	(-2,5;-2,0)	-3,6*	(-3,7;-3,4)
Cutaneous melanoma	2,3	3,2	5,8	6,6	7,5	8,2	9,0	9,8	+1,7*	(+0,9;+2,6)	-0,4	(-1,4;+0,6)
Prostate	114,5	112,0	115,6	121,0	117,5	106,3	94,7	81,9	-2,5*	(-2,9;-2,2)	-2,0*	(-2,8;-1,3)
Bladder	62,1	70,7	79,6	85,5	83,2	68,2	60,6	52,2	-2,7*	(-3,0;-2,3)	-2,6*	(-3,3;-1,8)
Kidney	15,5	17,3	23,9	30,2	33,7	31,2	29,9	30,0	-0,4*	(-0,8;-0,0)	-1,8*	(-2,2;-1,5)
Leukemia	32,7	35,7	40,2	42,2	41,6	38,9	38,4	38,1	-0,2	(-0,6;+0,2)	-2,5*	(-3,0;-2,0)
Hodgkin's lymphoma	6,8	6,8	5,7	4,5	3,4	2,6	2,3	2,2	-1,7*	(-3,2;-0,2)	-3,6*	(-5,3;-1,8)
Non-Hodgkin's lymphoma	12,4	11,3	17,0	24,0	29,7	33,1	32,5	28,8	-1,4*	(-2,1;-0,6)	-3,1*	(-4,5;-1,8)
Women												
All causes of death	3654,2	3274,1	2965,1	2604,2	2294,1	2031,7	1793,3	1533,4	-2,7*	(-3,0;-2,5)	-2,2*	(-2,4;-2,0)
All cancers, benign and malignant	671,0	646,1	662,7	680,8	678,0	637,6	614,9	585,9	-0,9*	(-1,0;-0,7)	-1,2*	(-1,5;-0,9)
Lip, oral cavity and pharynx	5,9	5,4	5,8	6,1	5,7	5,5	5,5	5,4	-0,2	(-1,2;+0,8)	+0,5	(-0,9;+1,8)
Esophagus	6,9	6,8	6,2	5,8	5,0	4,4	4,1	3,4	-2,2*	(-3,2;-1,2)	-1,7*	(-3,2;-0,1)
Stomach	127,1	101,2	86,4	74,2	58,8	46,0	38,4	32,6	-3,5*	(-4,0;-3,0)	-2,6*	(-3,4;-1,7)
Intestines, mainly colon and rectum	94,2	98,4	77,0	79,4	77,3	73,0	69,0	67,0	-0,9*	(-1,2;-0,7)	-1,4*	(-1,9;-0,8)
Liver	14,0	17,7	20,2	18,4	23,0	19,1	17,4	10,4	-5,3*	(-7,9;-2,6)	-7,7*	(-10,1;-5,2)
Pancreas	24,0	27,2	31,0	37,4	39,8	39,6	42,2	43,1	+0,9*	(+0,6;+1,2)	+1,0*	(+0,4;+1,6)
Larynx	2,2	2,1	2,1	1,9	1,8	1,4	1,4	1,1	-1,8	(-4,4;+0,8)	-1,1	(-3,2;+1,1)
Lung	33,3	38,6	44,9	52,6	57,7	61,1	63,1	65,3	+0,6*	(+0,4;+0,9)	+2,5*	(+2,1;+2,9)
Cutaneous melanoma	1,6	2,7	4,1	4,5	4,8	4,9	4,7	4,7	-0,3	(-1,4;+0,7)	-0,3	(-1,2;+0,7)
Breast	82,3	83,7	93,5	100,6	103,7	96,8	91,5	87,3	-1,1*	(-1,5;-0,7)	-2,1*	(-2,4;-1,7)
Ovary	16,8	18,0	22,2	28,1	29,8	27,4	26,9	27,2	0,0	(-0,6;+0,6)	-0,8*	(-1,5;-0,2)
Bladder	11,3	12,4	12,9	12,4	12,0	9,4	8,7	7,9	-1,9*	(-2,5;-1,2)	-1,2	(-3,2;+0,8)
Kidney	7,5	8,2	9,8	11,6	12,0	11,3	11,0	10,4	-0,9*	(-1,5;-0,3)	-2,4*	(-3,5;-1,4)
Leukemia	18,3	20,0	21,8	21,4	22,6	21,0	20,7	20,3	-0,4	(-0,9;+0,2)	-3,0*	(-3,9;-2,1)
Hodgkin's lymphoma	3,6	4,0	3,3	2,5	2,1	1,4	1,3	1,3	-0,8	(-2,4;+0,9)	-2,9*	(-5,7;-0,1)
Non-Hodgkin's lymphoma	6,9	6,8	10,0	14,7	19,7	22,6	22,4	18,5	-1,7*	(-2,7;-0,7)	-3,8*	(-4,9;-2,7)

*Significantly different from 0 (p < 0.05); *APC*: Average Percent Change.

In men, esophagus, larynx, and stomach cancer mortality rates decreased continuously throughout the whole period. Hodgkin lymphoma mortality declined after 1975-79. Cancers of the lip, oral cavity and pharynx, bladder, and prostate declined after 1985-89. Declines after 1990-94 were recorded for cancers of the liver, lung, and kidney (which leveled-off from 2000-03). After 1995-99 declines were observed for cancer of the intestines, and non-Hodgkin lymphomas. Conversely, increased trends were observed for cancer of the pancreas and for cutaneous melanoma.

In women, esophagus, larynx, and stomach cancers declined continuously throughout the entire period, while intestines cancer, and Hodgkin lymphoma declined after 1975-79. Lip, oral cavity and pharynx cancers decreased after 1985-84 (reaching a plateau from 1995-99 onwards). Cancers that declined after 1990-94 were liver, breast, ovary (that leveled-off since 1995-99), bladder, kidney, and leukemia (stable since 2000-03). Non-Hodgkin lymphoma declined after 1995-99. By contrast, lung and pancreatic cancer mortality rates increased throughout the whole period examined. Cutaneous melanoma increased up to 1995-99 and leveled-off thereafter.

Furthermore, between 1995 and 2008 in the elderly, cancer rates showed several statistically significant negative APCs. In elderly men the highest decreases were displayed by cancers of the liver, and larynx (APC = -4.6%/year), followed by cancer of the esophagus (APC = -3.7%/year), stomach (APC = -3.3%/year), lip, oral cavity and pharynx (APC = -2.8%/year), bladder (APC = -2.7%/year), prostate (APC = -2.5%/year), lung (APC = -2.2%/year), Hodgkin lymphomas (APC = -1.7%/year), non-Hodgkin lymphomas (APC = -1.4%/year), and intestines (APC = -0.8%/year). Increases were seen for pancreas carcinoma (APC = +0.6%/year), cutaneous melanoma (APC = +1.7%/year), and kidney cancer that leveled-off from 2000-03. In women the largest decreases were observed by cancer of the liver (APC = -5.3%/year) followed by cancer of the stomach (APC = -3.5%/year), esophagus (APC = -2.2%/year), bladder (APC = -1.9%/year), larynx (APC = -1.8%/year), non-Hodgkin lymphoma (APC = -1.7%/year), breast (APC = -1.1%/year), and kidney (APC = -0.9%/year). Increases were displayed by cancer of the pancreas (APC = +0.9%/year) and lung (APC = +0.6%/year). Lip, oral cavity and pharynx cancers, ovary, melanoma, Hodgkin lymphomas and leukemia showed stable trends.

By comparison with the other age groups, between 1995 and 2008 overall cancer mortality rates declined for people aged <40 years (-1.7%/year in men and -1.8%/year in women), and for middle aged (35-64 years) people (-2.6%/year in men and -1.2%/year in women) (Table 2) while rates in the very elderly (≥85 years) people increased in men (+0.6%/year) and decreased in women (-0.2%/year) (not shown). As compared to the elderly, middle aged men displayed higher decreases in cancer mortality for lung, kidney, and leukemia, while middle aged women showed higher decreases in cancer mortality for breast, leukemia and non-Hodgkin’s lymphomas. Conversely, middle aged women presented higher increases of lung cancer mortality than elderly women. The other APCs were similar in elderly and middle aged people.

## Discussion

Our investigation of cancer mortality trends among the Italian elderly population across approximately four decades (1970-2008) showed an increase of the absolute number of cancer deaths, but decreases in cancer mortality rates for several sites. These decreases reflected those observed in four leading cancers in men (stomach, bladder, prostate, and lung) and three leading cancers in women (stomach, bladder, and breast). Exceptions to these favorable trends were the increased mortality rates for lung cancer in women, cutaneous melanoma in men, and pancreatic cancer in both sexes. However, the general favorable trends of the majority of cancers were still modest over the same period of time when compared to the higher drops in mortality observed for diseases of the circulatory system and respiratory system, (Additional file 1: Table S1). As compared to middle aged people, the elderly displayed lower decreases for leukemia, lung and kidney cancers in men and for leukemia, non-Hodgkin’s lymphomas, and breast cancer. By contrast, middle aged women presented higher increases of lung cancer mortality than elderly women.

The downward trends observed for cancer mortality rates among Italian elderly between 1995 and 2008 were generally consistent with those reported over a comparable period of time in Japan (1998-2007), Canada (1995-2004), United States (1996-2005), and England, and Wales (1997-2006) [16].

Several reasons related to large scale changes in exposure to risk factors and to improvements in diagnosis and treatment may explain these trends. For instance, decreases in prevalence of smoking in men caused the decline of lung cancer incidence and mortality, and partially a decline in bladder, kidney, and larynx cancer [17,18]. The time shift observed for bladder cancer indicates, in addition to smoking, a reduction in exposures to occupational carcinogens [18]. Moreover, the decrease and subsequent plateau reached by kidney cancer indicates that obesity and/or hypertension inhibit further drops in rates of this cancer [19]. Decreases in alcohol consumption partly explain the reductions of cancers of the lip, oral cavity and pharynx, esophagus, and larynx [20]. In men, smoking reduction hampered the drop of lip, oral cavity and pharynx, and larynx cancers in the mid-1980s [20]. Changes in viral hepatitis prevalence rather than alcohol consumption seemed to explain the drop of liver cancer, as alcohol related cancers declined since the 1970s [21]. However, the complexity in differentiating primary and secondary liver cancer in death certificates requires some caution in the interpretation of these results. Reduction of stomach cancer mortality is generally attributed to a healthier diet, improved preservation of foods, and reduced prevalence of Helicobacter pylori [22]. Advancements in screening and early diagnosis and, consequently, effective therapy can chiefly explain the peak of breast cancer observed in the early 1990s. Ovarian cancer displayed a parallel peak to the one of breast cancer that leveled-off since the mid-1990s, a pattern that may be linked to delayed diagnoses [23]. Progresses in surgical techniques, adjuvant therapy, and declines in exposure to risk factors are likely explanations for the slight decrease in intestinal cancer over time [24]. Population-based screening for colorectal cancers was not widely available in Italy at the time of the study; therefore, it could not influence these trends, while drops in the mortality for prostate cancer since the mid-1980s are likely due to therapy advancements.

Although evidence-based data restricted to the elderly are scanty, it is generally accepted that overall improvements in anti-cancer treatment has contributed to reduce cancer mortality also in the elderly. An example is the early adoption of transurethral resection of the prostate as well as androgen blockage and radiotherapy for patient with locally advanced disease. Moreover, declines in mortality rates for prostate cancer have been affected also by chemotherapy with Docetaxel [25].

Pancreatic cancer mortality increased since the 1970s, leveled-off in correspondence to the peak of lung cancer in mid-1990s, to increase thereafter, maybe, in association with the increase of diabetes prevalence [26]. Cutaneous melanoma is directly linked to excessive sun exposure. The level-off in women, but not in men, indicates changes in exposure rather than selective therapy advancements [27]. Finally, therapy advancements were associated to the peak of Hodgkin lymphoma mortality in the mid-1970s. Compared with young patients, the elderly are at an advanced stage, have lower performance status, and generally have a more aggressive biology of the disease and poor outcome [28]. The decrease in mortality for non-Hodgkin lymphoma at the end of 1990s is attributable to improved therapies like the introduction of Rituximab [29]. Death certificates do not allow the distinction of various types of leukemia; therefore, the observed trends are difficult to interpret.

Cancer death certifications data have some limitations in accuracy and completeness that should be made clear. Firstly, the primary site of particular cancers is not always ascertained by means of death certificates. In accordance with such drawbacks, some sites were pooled together: lip, oral cavity and pharynx, colon and rectal cancers, non-Hodgkin lymphomas, and leukemias. Moreover, before ICD-10 use, primary liver cancer was not always distinguishable from metastases of other cancers. These limitations were taken into account in the interpretation of time trends. Secondly, cancer and comorbidities are extremely common in elderly people and the percentage of histologically confirmed cancers is generally lower among them than in younger age groups. Thus, the identification of the true underlying cause of death may be difficult, with occurrence of both false positive and false negative errors. Between 1970 and 2008, three different ICD coding rules were used to indicate the underlying cause of death and the criteria for malignancy. The changes in the classification of tumors were overcome by recoding diagnoses according to ICD-10. The overall increased precision should have inflated mortality rates over time; however, in this study, the main groups of diseases showed declines. Thirdly, the accuracy of mortality data derived from death certificates depends from coding practices. In Italy, the coding of the cause of deaths is conducted at national level according to strict guidelines, also by means of an automated system, thus limiting the possibility of differential practices influencing death certification procedures.

Some points can be drawn from the trends of cancer mortality in the elderly in the last four decades. Firstly, preventive strategies, smoking in the first place, apply well to the elderly. Cancer prevention must be further promoted and targeted to this specific population. Secondly, the elderly generally present high prevalence of slowly growing cancers, a life expectancy, and a quality of life compatible with a benefit from screening or early diagnosis However, they are frequently excluded from these benefits. Thirdly, the elderly may be unable to access care that is otherwise considered standard for an adult population.

## Conclusions

In conclusion our study, focused on the whole Italian elderly population, showed favorable trends in mortality rates from 1970 to 2008 for many cancers, although in absolute terms, the number of cancer deaths doubled. However, further efforts in prevention, applicability of screenings, and access to care need to be reconsidered as the Italian population ages.

## Competing interests

The authors declare that they have no competing interests.

## Authors’ contributions

EB and DS performed all analyses and produced the primary manuscript; SB and MP participated in the acquisition of data; All authors read, participated in discussions of appropriate groups for analysis and interpretation, and approved the final manuscript.

## Pre-publication history

The pre-publication history for this paper can be accessed here:

http://www.biomedcentral.com/1471-2407/12/443/prepub

## Supplementary Material

Additional file 1 **Table 3. **Time trends of main causes of death among elderly and middle aged populations; Italy, 1970-2008. Click here for file
